# Type I interferon receptor knockout mice as models for infection of highly pathogenic viruses with outbreak potential

**DOI:** 10.24272/j.issn.2095-8137.2017.052

**Published:** 2018-02-09

**Authors:** Gary Wong, Xiang-Guo Qiu

**Affiliations:** 1 Shenzhen Key Laboratory of Pathogen and Immunity, State Key Discipline of Infectious Disease, Shenzhen Third People’s Hospital, Shenzhen Guangzhou 518020, China; 2 Key Laboratory of Pathogenic Microbiology and Immunology, Institute of Microbiology, Chinese Academy of Sciences, Beijing 100101, China; 3 Special Pathogens Program, National Microbiology Laboratory, Public Health Agency of Canada, Winnipeg, Manitoba R3E 3R2, Canada; 4 Department of Medical Microbiology and Infectious Diseases, University of Manitoba, Winnipeg, Manitoba R3E 0J9, Canada

**Keywords:** Ifnar, Mice, Animal model, *Flavivirus*, *Filovirus*, *Arenavirus*, *Bunyavirus*, *Henipavirus*, *Togavirus*

## Abstract

Due to their inability to generate a complete immune response, mice knockout for type I interferon (IFN) receptors (*Ifnar^–/–^*) are more susceptible to viral infections, and are thus commonly used for pathogenesis studies. This mouse model has been used to study many diseases caused by highly pathogenic viruses from many families, including the *Flaviviridae*, *Filoviridae*, *Arenaviridae*, *Bunyaviridae*, *Henipaviridae*, and *Togaviridae*. In this review, we summarize the findings from these animal studies, and discuss the pros and cons of using this model versus other known methods for studying pathogenesis in animals.

## INTRODUCTION

Outbreaks of infectious diseases amongst the human population have been documented for thousands of years. The earliest on record was the Plague of Athens between 429–426 B.C.. Caused by an unknown pathogen, the outbreak killed over 75 000 people (Littman, 2009). Epidemics that have occurred since are too numerous to list thoroughly in this review, but include multiple instances of plague (*Yersinia pestis*) that devastated populations of Europe, Asia and North Africa with hundreds of millions of deaths. The most infamous epidemic of plague was the “Black Death” during 1346–1350 (Haensch et al., 2010), in which an estimated 30%–60% of the population was wiped out. Viral outbreaks including those caused by smallpox, measles and viral hemorrhagic fevers in various locations worldwide have impacted tens of millions (CDC 2017; Moss & Griffin, 2012; Thèves et al., 2016). In the 20^th^ century, three major influenza pandemics (H1N1 during 1918, H2N2 during 1957–1958 and H3N2 during 1968–1969) have killed over 75 million people combined (Johnson & Mueller, 2002; Kilbourne, 2006). As we enter the 21^st^ century, the death toll from outbreaks of infectious diseases has decreased dramatically, and the highest numbers of fatalities were from the 2014–2016 Ebola virus outbreak in West Africa (over 11 000 deaths) (WHO, 2016a) and the 2009 H1N1 influenza pandemic (over 18 000 deaths) (WHO, 2010). Considerable advances and deployment of prophylactics, therapeutics, rapid point-of-care diagnostics and surveillance have limited the negative impacts from outbreaks in many parts of the world and saved many lives that would otherwise have been lost. However, outbreaks of re-emerging infectious diseases have been occurring with ever increasing frequency in recent years, and there is still much to do in the war against infectious diseases. ^1^

The use of animals to study pathogenesis, as well as test potential vaccines and drugs, have played a big role in accelerating the most promising compounds through the pre-clinical process before testing in clinical trials. Non-human primates (NHPs), the closest relative species to humans, are considered the gold standard animal model for many infectious diseases (Safronetz et al., 2013) because these animals recapitulate multiple aspects of human disease, and thus any experimental results are expected to have high translatability and applicability to humans. However, NHPs are costly to acquire, difficult to handle, and require specialized facilities to house and provide husbandry (Coleman, 2011), and are unaffordable for many research laboratories. To address this, smaller animals have been used for many preliminary studies and screens of candidate vaccines and drugs. For instance, the domestic ferret (*Mustela putorius furo*) was used to study many member viruses belonging to the order *Mononegavirales* (Enkirch & von Messling, 2015).

Mice are an ideal species for studying human infectious diseases. The immune systems of mice and humans are often sufficiently similar that they can be infected with the same pathogens (Buer & Balling, 2003). For example, immunocompetent wild-type mice are susceptible to infections with a number of influenza virus subtypes (Belser et al., 2010; Driskell et al., 2010; Gubareva et al., 1998; Xu et al., 2013), severe acute respiratory syndrome coronavirus (SARS-CoV) (Channappanavar et al., 2016) and Rift Valley fever virus (RVFV) (Smith et al., 2010), and outbreaks with these pathogens can be rapidly and easily studied. Unfortunately, wild-type mice are not susceptible to many other pathogens with outbreak potential, and thus alternative strategies are needed. Mice lacking the type I interferon (IFN) receptor (*Ifnar^–/–^*) were generated in 1994 (Muller et al., 1994). While these transgenic mice do not show any overt abnormalities by six months of age and are fertile, the animals are entirely unresponsive to the effects of type I IFNs. Ablated immune responses in *Ifnar^–/–^* mice were observed after challenge with Vesicular stomatitis virus, Semliki Forest virus, vaccinia virus, or lymphocytic choriomeningitis virus, and the knockout animals showed enhanced susceptibility resulting in either higher viral organ titers, or death at lower doses compared to wild-type mice (Muller et al., 1994).

Since the type I IFN response plays such an important role in innate and adaptive immunity against viral infections (McNab et al., 2015), the *Ifnar^–/–^* mice, which are available in many backgrounds, have since been used to study many highly pathogenic viruses. In this review, we summarize the results of using *Ifnar^–/–^* mice to study selected pathogens from the *Flaviviridae*, *Filoviridae*, *Arenaviridae*, *Bunyaviridae*, *Henipaviridae*, and *Togaviridae* families, focusing on member viruses that either have, or may have the potential to cause large scale outbreaks in the future. 

## *FLAVIVIRIDAE* 

The family *Flaviviridae* contains many member viruses which are highly pathogenic to humans and/or have high outbreak potential. West Nile fever, Dengue fever, Yellow fever, Japanese encephalitis, and Zika fever are all mosquito-borne diseases, caused by West Nile virus (WNV), Dengue virus (DENV), Yellow fever virus (YFV), Japanese encephalitis virus (JEV) and Zika virus **(**ZIKV), respectively.

### West Nile virus (WNV)

Found in temperate and tropical regions, WNV is maintained in a mosquito-bird-mosquito cycle in nature, with humans as incidental hosts. WNV was firstly identified in Uganda in 1937, but the majority of infections (~80%) caused only mild disease or were asymptomatic (WHO, 2011). In case of symptomatic disease, fever, headache, fatigue, muscle pain, nausea, vomiting and rash is observed. Less than 1% of cases are neuroinvasive, in which patients present high fever, stupor, disorientation, coma, tremors, convulsions, muscle weakness, and paralysis (WHO, 2011). Large outbreaks of WNV occurred sporadically throughout the decades, but during 1996 WNV re-emerged in Romania and caused 393 confirmed infections, in which 352 patients had manifestations in the central nervous system (Tsai et al., 1998). Subsequent epidemics of WNV with high rates of neuroinvasive disease was then noted in Morocco in 1996, Tunisia in 1997, and large outbreaks in Italy and Israel in 1998 (Hubálek & Halouzka, 1999). At present, WNV is endemic in Africa, Asia, Europe, Australia, and has spread into Canada and the United States (Chen et al., 2013). An outbreak of WNV in 2012 in the United States claimed 286 lives (Murray et al., 2013).

Wild-type 129Sv/Ev mice infected subcutaneous (SC) with 10^2^ plaque forming units (pfu) of WNV showed 62% mortality and died at a mean time to death of 11.9±1.9 days post infection (dpi), with no clinical signs observed until 8 dpi (Samuel & Diamond, 2005). In their study, Samuel et al. challenged 8–10 week old *Ifnar^–/–^* mice (129Sv/Ev background) with 10^0^, 10^1^ or 10^2^ pfu or WNV strain 3000.0259 via footpad (SC) inoculation. Regardless of dose, all mice showed severe clinical symptoms by 3 dpi, including hunched posture, ruffled fur and reduced activities. Death occurred within 12–48 hours after the onset of symptoms, and the mean time to death was 3.8±0.5 dpi for *Ifnar^–/–^* mice in the 10^2^ pfu group (Samuel & Diamond, 2005). Live infectious virus can be found in the muscle, heart, lung, kidney, liver, but not in the pancreas. Additionally, challenge of *Ifnar^–/–^* mice (C57BL/6 background) also showed 100% lethality and a mean time to death of 3.4±0.5 dpi (Samuel & Diamond, 2005).

### Dengue virus (DENV)

DENV is widespread in the temperate and tropical regions of the world, and each year approximately 50–150 million people are infected (Bhatt et al., 2013), with over 10 000 deaths (Stanaway et al., 2016). Symptoms of Dengue fever include a high fever, headache, vomiting, muscle and joint pains, and skin rash. Severe cases of disease is usually associated with secondary infection with heterologous types of DENV (Halstead, 1988), and can develop into Dengue hemorrhagic fever (with hemorrhage, thrombocytopenia and blood plasma leakage), or into Dengue shock syndrome, both of which are potentially fatal (Kularatne, 2015).

Infection of 129Sv/Ev mice with 10^8^ pfu DENV-2 via the intravenous (IV) route resulted in 87% survival (26 out of 30 mice), and inoculation with 4.4×10^4^ pfu DENV-1 via IV resulted in 93% survival (40 out of 43 mice) (Shresta et al., 2004). In their study, Shresta et al. challenged 5–6 week old *Ifnar^–/–^* mice (129Sv/Ev background) with DENV-2 strain PL046 (*n*=12) and DENV-1 strain Mochizuki (*n*=16) at the same doses and inoculation routes. While no lethality was observed, sera and major organs harvested from infected mice at 3 and 7 dpi showed the presence of virus in all sera, liver, spleen, and lymph node samples, as well as some brain and spinal cord samples (Shresta et al., 2004). Interestingly, mice deficient for the type I and II IFN receptors (AG129) showed uniform death from DENV-2 infection with animals dying between 7–30 dpi, and DENV-1 as well with mice succumbing to disease between 7–14 dpi (Shresta et al., 2004).

### Yellow fever virus (YFV)

YFV is endemic in tropical areas of Africa and South America (WHO, 2016b), when the virus was introduced via the slave trade during the 17^th^ century. Many infections are symptomatic, but if clinical symptoms appear, they include fever, chills, appetite loss, nausea, muscle pains, and headaches. A small percentage (~15%) of cases will go on to develop more severe disease including jaundice, dark urine, vomiting and abdominal pain. Hemorrhage from the mouth, nose, eyes or stomach may occur and 50% of patients with these symptoms succumb to disease (WHO, 2016b). YFV was responsible for ~127 000 severe infections and 45 000 deaths in 2013 (WHO, 2016b), with increased incidence over the past decades, and the risk of an outbreak in urban centers is a serious public health threat (Barrett & Higgs, 2007).

Inoculation of wild-type 129 mice SC in each rear footpad with 10^4^ pfu of YFV did not result in any weight loss or death (Meier et al., 2009). In their study, Meier et al. (2009) challenged 3–4 week old *Ifnar^–/–^* mice (129 background) with YFV strains Asibi or Angola73 under the same conditions. The mice were shown to be susceptible to the challenge, with death occurring between 7–9 dpi. Additionally, the mice developed viscerotropic disease with virus dissemination to the visceral organs, spleen and liver, in which severe damage of the organs can be observed with gross pathological examination and hematoxylin/ eosin staining. Elevated levels of MCP-1 and IL-6 in these organs are suggestive of a cytokine storm (Meier et al., 2009).

### Japanese encephalitis virus (JEV)

Japanese encephalitis is an acute disease of the central nervous system in humans. Infected patients develop a febrile illness with headaches vomiting and diarrhea, as well as reduced levels of consciousness, seizures, and photophobia. Severe encephalitis occurs later in the disease course and is associated with a higher frequency of seizures, resulting in coma and death (Ghosh & Basu, 2009). Mental retardation may develop in the patient. JEV is endemic in large parts of Asia and the Pacific, and 30 000–50 000 infections (Solomon, 2006), including up to 15 000 deaths (Ghosh & Basu, 2009), are reported yearly. It is estimated that approximately 25%–30% of cases are fatal, but 50% result in permanent neurological sequelae (Ghosh & Basu, 2009).

Inoculation of wild-type 129 mice via the SC route with 10^0^, 10^2^, 10^4^ or 10^6^ pfu of JEV strain JaOArS982 resulted in between 10%–40% survival, but the difference was not statistically significant and deaths were not dose-dependent (Aoki et al., 2014). In their study, Aoki et al. repeated the SC infection in 5–6 week old *Ifnar^–/–^* mice (129 background) at the same doses. The mice were found to be very susceptible to the challenge, with uniform, dose-dependent death occurring at 64, 80, and 96 hours after infection for the 10^6^, 10^4^ and 10^2^ pfu groups, respectively. In the 10^0^ group, 90% mortality rate was observed, and the animals died at 120 hours after infection. Live JEV could be detected in the spleens and brains of infected animals, with peak titers at 48 hours (Aoki et al., 2014).

### Zika virus (ZIKV)

First isolated in 1947 from an infected monkey in Uganda and re-isolated from *Aedes* mosquitoes in the same area during 1948 (Dick et al., 1952), ZIKV infections in humans have sporadically occurred in Africa and Asia, but in 2007 the virus continued spreading, causing outbreaks in small island countries located in the Pacific Ocean, such as Yap Island (Duffy et al., 2009), French Polynesia (Cao-Lormeau et al., 2014) and Easter Island (Tognarelli et al., 2016). In early 2015, an epidemic of ZIKV infections, originating from Brazil, spread through most of North and South America and the Caribbean with tens of thousands of people over 80 countries infected (WHO, 2017), as well as thousands of imported cases from travelers returning to their home countries after visiting outbreak areas. The epidemic was declared over by the World Health Organization (WHO) on November 2016 (WHO, 2017), but many countries are still dealing with the long-term impact of ZIKV infections. Infections of ZIKV are typically asymptomatic, but if present they are mild in nature and includes fever, joint pain, maculopapular rash, and bloodshot eyes (Simpson, 1964). While no deaths have been reported from ZIKV infections, mother-to-child transmission during pregnancy may result in congenital Zika syndrome with abnormalities in the central nervous system (microcephaly, intellectual development, seizures and vision impairment) (Boeuf et al., 2016). ZIKV infections in adults is associated with Guillain–Barré syndrome (Frontera & da Silva, 2016). Distinct from other flavivirus infections, sexual transmission of ZIKV from male-to-male (Deckard et al., 2016), male-to-female (D'Ortenzio et al., 2016; Hills et al., 2016) and female-to-male (Davidson et al., 2016) have been documented. 

Infection of wild-type 129Sv/Ev mice SC with 10^6^ pfu of ZIKV MP1751 did not result in any observable clinical symptoms or histological changes, despite the virus being detected at low levels in the blood, spleen and ovaries (Dowall et al., 2016). In their study, Dowall et al. challenged 5–6 week old *Ifnar^–/–^* mice (129Sv/Ev background) under the same conditions as above, and showed that all animals succumbed to disease at 6 dpi with 20% body weight loss. High levels of virus could be detected by RT-qPCR at 3 and 7 dpi in the blood, spleen, brain, ovary and livers of these animals. Pathology studies show that inflammatory as well as degenerative changes could be seen in the brains of infected *Ifnar^–/–^* mice (Dowall et al., 2016). In another study, Lazear et al., (2016) inoculated 5–6 week old *Ifnar^–/–^* mice (C57BL/6 background) with 10^2^ pfu of ZIKV strain H/PF/2013 or MR766 via the SC route in the footpad. The results show that *Ifnar^–/–^* mice all died within 8–10 dpi after challenge with H/PF/2013, and 80% death with MR766, with death between 9–13 dpi. Additionally, an SC challenge with 10^3^ focus forming units (ffu) of ZIKV strain Dakar 41671, 41667 or 41519 in *Ifnar^–/–^* mice results in uniform death by 6 dpi (Lazear et al., 2016). In a third study, Rossi et al. (2016) inoculated 3-, 5- and 11-week old *Ifnar^–/–^* mice (C57BL/6 background) with 1×10^5^ pfu of ZIKV FSS13025 via the SC route. The results showed 100% lethality in 3-week old animals with death occurring at 6–7 dpi, but only 50% death in 5-week old animals and no deaths in 11-week old animals (Rossi et al., 2016), indicating that the disease caused by ZIKV infection in these animals is age-dependent.

## *FILOVIRIDAE* 

### Ebola virus (EBOV), Sudan virus (SUDV), Reston virus (RESTV), Tai Forest virus (TAFV), Marburg virus (MARV) and Ravn virus (RAVV)

The family *Filoviridae* consists of many member viruses, including EBOV, SUDV, RESTV, TAFV and MARV, among others. With the exception of RESTV, all filoviruses are pathogenic in humans and infected patients initially present with fever, sore throat, muscular pain, headaches, vomiting, and diarrhea. As the infection develops, a rash is observed along with decreased organ function (especially liver and kidneys). Hemorrhage, shock and eventually multiple organ failure results in the death of the patient (Bradfute et al., 2012). Outbreaks of filovirus disease in humans are sporadic and unpredictable, and typically localized geographically to sub-Saharan Africa, but imported cases have occurred in the past to Europe and North America (CDC, 2014, 2017b). The case fatality rate (CFR) of EBOV and MARV can reach up to 90%, whereas SUDV is ~50%. Only one case of TAFV has been recorded, in which the patient fell ill but survived infection (Formenty et al., 1999). The CFR of RAVV cannot be estimated accurately since the only large scale outbreak during 1998–2000 in the Democratic Republic of the Congo (128 deaths out of 154 cases) was due to the simultaneous co-circulation of RAVV and MARV (Bausch et al., 2003, 2006).

Infection of wild-type adult immunocompetent 129 mice does not result in disease or death (Bray, 2001). Bray (2001) then inoculated 8–16 week old *Ifnar^–/–^* mice (129 background) with 1 000 pfu of EBOV, SUDV, RESTV, TAFV as well as MARV via intraperitoneal (IP) route. The results show that *Ifnar^–/–^* mice succumbed to infection with the Mayinga isolate of EBOV, with a mean time to death of 5.4 dpi, but resistant to the Kikwit isolate of EBOV. SUDV strain Boneface produced uniformly lethal infection with a mean time to death of 6.3 dpi, but RESTV and TAFV infections did not result in death of the *Ifnar^–/–^* mice. Infection with RAVV and MARV produced 100% and 67% lethal infections, with a mean time to death of 6.0 and 8.5 dpi, respectively. Additionally, a SC challenge of the Mayinga isolate of EBOV to *Ifnar^–/–^* mice was shown to be fully lethal with a mean time to death of 7.3 dpi (Bray, 2001). In another study, 6–9 week old *Ifnar^–/–^* mice (129 background) were challenged with an aerosol dose of MARV between 10^2.8-5.8^ 50% tissue culture infective doses (TCID_50_), EBOV between 10^0-2^ TCID_50_ of EBOV, or SUDV at 10^4.8^ TCID_50_. All animals challenged with MARV succumbed to disease at a mean time to death of 11–13 dpi, whereas EBOV-infected mice died at an average of 8 dpi. Clinical symptoms such as lethargy, weight loss and piloerection were observed prior to death. Although symptoms such as anorexia were observed in SUDV-infected mice from 7–11 dpi, all infected mice survived and returned to their pre-challenge weights by the conclusion of the experiment (Lever et al., 2012).

## *ARENAVIRIDAE* 

### Lassa virus (LASV)

Lassa fever is prevalent in the West African countries of Nigeria, Liberia, Sierra, Leone, Mali, Ghana, and Guinea, in which 300 000–500 000 cases are reported yearly, including 5 000 deaths (CFR ~1%) (Ogbu et al., 2007); however, the CFR from nosocomial outbreaks can reach as high as 65% (Fisher-Hoch et al., 1995). Carried by the multimammate rat (*Mastomys natalensis*), most infected patients are asymptomatic, but if illness occurs the initial presentation includes fever, weakness, headaches, vomiting, and muscle pains. In advanced disease, haemorrhaging, encephalopathy, shock and organ failure is observed (Schmitz et al., 2002).

Wild-type 129S1SvImJ mice are naturally resistant to infection with LASV (Yun et al., 2012). In their study, Rieger et al. infected 8–12 week old *Ifnar^–/–^* mice (129/Sv background) with 10^3^ ffu of LASV strains Josiah, AV, BA366 and Nig04-10 via the IV route. No deaths were observed with the mice, but the peak of viremia (10^4.5-6^ ffu/mL of blood) was detected at 8 dpi and still not fully cleared by 21 dpi. Weight loss of approximately 15% by 8 dpi was observed, along with elevation of liver enzymes AST and ALT. Other findings include the presence of high levels (up to 10^7^ ffu/g of tissue) of live LASV in the lung, kidney, heart, spleen, brain and liver infected animals at 9–10 dpi (Rieger et al., 2013). Thus, the results support the establishment of productive LASV infection in *Ifnar^–/–^* mice.

## *BUNYAVIRIDAE* 

### Crimean-Congo hemorrhagic fever virus (CCHFV)

Crimean-Congo hemorrhagic fever is caused by infections with CCHFV, which was first reported in the 1940s, but a study suggests that the virus may have been present since 1 500–1 100 B.C. (Carroll et al., 2010). Initial symptoms of CCHFV infection include fever (over 39.0 ℃), muscle pains, fatigue, dizziness, vomiting, and diarrhea (Whitehouse, 2004). Advanced CCHFV infections are characterized by more severe symptoms including liver failure, petechiae as well as gastrointestinal and cerebral hemorrhage resulting in death (Whitehouse, 2004). The CFR can vary widely: it was reported to be 5% during an outbreak in Turkey (Kubar et al., 2011), but 60% during another outbreak in the UAE (Schwarz et al., 1996). Spread by *Hyalomma* ticks, cases of CCHFV infections in humans has been reported in western Asia, Eastern Europe, the Middle East, as well as South Africa, although the geographical distribution of the *Hyalomma* vector is widespread and encompasses all of Africa, as well as European and Asian regions south of the N50^°^ latitude (WHO, 2008). Approximately 50 cases are reported per year worldwide, but over 200 cases were reported during 2003–2004 (Messina et al., 2015).

Infection of wild-type 129 Sv/Ew mice with CCHFV at high doses results in the establishment of an infection that is rapidly cleared from the kidney, brain, heart and blood within 3 dpi, and clearance from the liver and spleen by 11 dpi, but no clinical signs or mortality (Bereczky et al., 2010). In their study, Bereczky et al. challenged 7–10 week old *Ifnar^–/–^* mice (129 Sv/Ew background) IP with 10^1^–10^6^ ffu per animal of CCHFV strain IbAr 2000. Symptoms including laboured breathing were observed between 42–70 hours after infection and uniform death was observed at all doses. The highest viral loads in *Ifnar^–/–^* mice were observed at 2 dpi in the spleen and liver (over 10^10^ viral RNA copies/g of tissue), but could also be detected in blood, as well as other major organs including the kidney, brain and heart (Bereczky et al., 2010). In another study, Zivcec et al. infected 6–12 week old *Ifnar^–/–^* mice (C57BL/6 background) with 10^4^ TCID_50_ of CCHFV strain IbAr 2000 via the IP, intramuscular (IM), intranasal (IN) and SC routes, and showed that all animals died with an average time to death of 4±0, 5.2±0.6, 7±0 and 4.6±0.2 dpi, respectively (Zivcec et al., 2013). Thrombocytopenia, coagulopathy, strong pro-inflammatory responses were observed in these animals. Live CCHF of up to 10^4^ TCID_50_/mg of tissue could be detected in the blood, lymph node and various major organs (Zivcec et al., 2013). In contrast, infection of wild-type C57BL/6 mice with CCHFV did not result in any pathology (Zivcec et al., 2013).

### Severe fever with thrombocytopenia syndrome virus (SFTSV)

Severe fever with thrombocytopenia is a newly recognized disease in rural areas of northeastern and central China, with several cases in Japan and South Korea (Promedmail, 2013). Caused by SFTSV, the transmission route of the virus is still unknown, but most likely involves arthropod vectors or animal hosts since the virus has been detected in ticks collected from domestic animals (Tian et al., 2017), and the animals (i.e., goats, cattle and dogs) also have high levels of SFTSV-specific antibodies (Jiao et al., 2012). Patients infected with SFTSV present with fever, vomiting, diarrhea, thrombocytopenia, leucopenia, and increased liver enzyme levels, in which severe cases of SFTSV eventually result in multiple organ failure resulting in death (Yu et al., 2011). The fatality rate amongst hospitalized patients can be up to 30%, and hundreds of cases are reported annually in China (Liu et al., 2015). 

Infection of wild-type mice (BALB/c, C57BL/6) results in limited weight loss but the animals do not succumb to disease (Chen et al., 2012; Jin et al., 2012). In one study, Liu et al. (2014) infected 6–10 week old *Ifnar^–/–^* mice (129/Sv background) SC with 10^6^ ffu of SFTSV strain YL-1. The mice were highly susceptible to challenge, with all mice appearing ill by 3 dpi, resulting in death between 3–4 dpi. Blood and major organs (brain, heart, kidney, intestine, liver, lung and spleen) were collected from infected *Ifnar^–/–^* mice daily, and results showed high levels of virus replication with systemic spread to all organs. In particular, the spleen and intestine had the highest peak virus titers at death (Liu et al., 2014). In another study, Matsuno et al. infected 6–12 week old *Ifnar^–/–^* mice (C57BL/6 background) with either a high dose (10^5^ TCID_50_ per animal) or a low dose (10^2^ TCID_50_ per animal) of SFTSV strain SD4 via the intradermal (ID), IP, IM or SC routes. The results showed that the *Ifnar^–/–^* mice were susceptible to infection via all routes, with animals succumbing to death at 4 and 6 dpi in the high and low dose groups, respectively (Matsuno et al., 2017). 

## *HENIPAVIRIDAE* 

### Hendra virus (HeV) and Nipah virus (NiV)

HeV was discovered in 1994 as the etiologic agent that caused an acute respiratory disease in horses in Australia with sporadic but lethal transmission to humans, with one fatal case developing pneumonitis, respiratory and renal failure, arterial thrombosis, and eventually cardiac arrest seven days after admission (Selvey et al., 1995). HeV currently still poses a threat to Australian livestock, and the CFR is estimated to be 60% for humans and 75% for horses (Field et al., 2011). NiV was discovered in 1999 in Malaysia with spread to neighbouring Singapore, resulting in 100 deaths from 257 human cases (CDC, 1999a). Patients typically present with respiratory problems and fever, as well as encephalitis with symptoms of headache, drowsiness, disorientation and confusion, rapidly progressing to coma. Since then, outbreaks of NiV have caused severe encephalitis in Bangladesh and India, with a CFR of ~75% (Lo & Rota, 2008). Pigs are susceptible to infection and act as amplifying hosts to humans (CDC, 1999b). Fruit bats are the natural reservoir for both viruses (Halpin et al., 2011). 

Wild-type mice are only susceptible to HeV or NiV infection if the virus is administered via the intracranial (IC) route, but not through any other types of inoculations (Dhondt et al., 2013). In their study, Dhondt et al. infected 3–18 week old *Ifnar^–/–^* mice (C57BL/6 background) IP with 10^6^ pfu of HeV. It was observed that while the infection was fully lethal in 3-week old mice, the susceptibility decreased with increasing age and the same dose of HeV in 18-week old mice only resulted in 50% mortality. The moribund mice died between 7–13 dpi. For NiV strain UMMC1, 4–12 week old *Ifnar^–/–^* mice (C57BL/6 background) were infected IP with increasing dosages from 100–10^6^ pfu. The mice were found to be uniformly susceptible with deaths between 6–9 dpi in the 10^6^ pfu group, and the LD_50_ was calculated to be 8×10^3^ pfu in *Ifnar^–/–^* mice. Infected mice with both viruses first showed behavioural changes including agitation, edginess and no grooming. Neurological symptoms were observed with advanced disease including tilted head and paralysis. A weight loss of approximately 15%–25% was observed 1–2 days before death and found to be a good predictor of mortality (Dhondt et al., 2013).

## *TOGAVIRIDAE* 

### Venezuelan equine encephalitis virus (VEEV)

Venezuelan equine encephalitis was first identified in Venezuela in 1938, and outbreaks of the causative agent, VEEV, have occurred mostly in Central and South America, but the United States have also reported cases (Weaver et al., 2004). A mosquito-borne virus (Beaman & Turell, 1991), VEEV can infect and amplify in equine species, resulting in encephalitis as well as progressive disorders in the central nervous system. Transmission of the virus to humans via the mosquito vector can result in the patient presenting with malaise, fever, headache and encephalitis (Weaver et al., 2004). The CFR is estimated to be 0.7%–1% (Weaver et al., 1996), but permanent neurological damage have been noted with survivors of VEEV-induced encephalitis (León et al., 1975).

Infection of wild-type mice with a virulent VEEV strain (V3000) results in death at approximately 8.3±0.5 dpi, but infection with an attenuated VEEV strain (V3032) does not result in mortality (Schoneboom et al., 2000). In their study, Schoneboom et al. infected 8–12 week old *Ifnar^–/–^* mice (129Sv/Ev background) with 1×10^3^ pfu of VEEV either a virulent (V3000) or attenuated strain (V3032) SC into the left rear footpad. Within 18–20 hours of infection with either virus, the mice displayed hunching, ruffled fur and appeared lethargic. Advanced disease includes convulsions and prostration resulting in death. The mean time to death was 1 dpi for both VEEV strains, and high levels of live VEEV could be detected in the sera and brains of moribund *Ifnar^–/–^* mice (Schoneboom et al., 2000).

### Chikungunya virus (CHIKV)

First isolated in Tanzania in 1952 and carried by *Aedes* mosquitoes, CHIKV infections in humans result in Chikungunya fever, which is a severe illness in humans characterized by fever, headache, myalgia, rash, and acute as well as persistent arthralgia (Burt et al., 2012). Despite considerable morbidity, the CFR is estimated to be 0.1%, with those older than 65 and/or underlying medical problems to be most at risk of death (Caglioti et al., 2013). CHIKV outbreaks occurred mostly in central/southern Africa and southeast Asia during the 1960s–2000s (Powers & Logue, 2007), but in recent years large-scale outbreaks have been reported on the island of Reunion (Roth et al., 2014), India (Pialoux et al., 2007) and also the Americas (Staples & Fischer, 2014), in which millions of infections were reported.

Infection of wild-type adult mice with 10^6^ pfu of CHIKV does not result in mortality (Couderc et al., 2008). In their study, Couderc et al. infected adult *Ifnar^–/–^* mice (129s/v) via ID with 20 pfu of CHIKV (Couderc et al., 2008) and found that the mice died within an average of 3±0.2 dpi, with an LD_50_ of 3 pfu. Infectious virus could be detected in the liver within 16 hours after infection and abundantly detected in the muscles, joints, skin, brain, liver, spleen and sera by 3 dpi. Another study by Pal et al. infected 6–8 week old *Ifnar^–/–^* mice (C57BL/6 background) SC in the footpad with 20 ffu of CHIKV, and found that all mice died within 4 dpi, but that these animals did not develop the arthritis observed in humans (Pal et al., 2013).

## SUMMARY

Immunocompromised *Ifnar^–/–^* mice have been shown to be a very good alternative small animal model for highly virulent pathogens that do not cause disease in immunocompetent mice. In this review, we described the different parameters and results from experimental infection of *Ifnar^–/–^* mice with various pathogens (Table 1). It is obvious that the advent of *Ifnar1^–/–^* mice undoubtedly constituted a major step forwards in allowing researchers to easily and rapidly study the pathogenesis of clinical isolates during a potential outbreak situation, as these animals are more susceptible to viral infections (Table 2).

**Table 1:** Summary of experimental parameters and results of *Ifnar^–/–^* mice challenged with various outbreak viruses

Pathogen	Strain	Age (weeks) and background	Challenge dose	Challenge route	Death rate	Mean time to death, or range	References
WNV	3000.0259	8–10, 129Sv/Ev	10^0^ pfu	SC	100%	Not provided	Samuel & Diamond, 2005
			10^1^ pfu			Not provided	
			10^2^ pfu			3.8±0.5 days	
		8–10, C57BL/6	10^2^ pfu	SC	100%	3.4±0.5 days	
DENV-2	PL046	5–6, 129Sv/Ev	10^8^ pfu	IV	0%	N/A	Shresta et al., 2004
DENV-1	Mochizuki		4.4×10^4^ pfu			N/A	
YFV	Asibi	3–4, 129	10^4^ pfu	SC	100%	7–9 days	Meier et al., 2009
	Angola73					7–8 days	
JEV	JEV JaOArS982	5–6, 129	10^0^ pfu	SC	90%	120 hours	Aoki et al., 2014
			10^2^ pfu		100%	96 hours	
			10^4^ pfu			80 hours	
			10^6^ pfu			64 hours	
ZIKV	MP1751	5–6, 129Sv/Ev	10^6^ pfu	SC	100%	6 days	Dowall et al., 2016
	H/PF/2013	5–6, C57BL/6	10^2^ pfu	SC		8–10 days	Lazear et al., 2016
	MR766					9–13 days	
	Dakar 41671		10^3^ ffu			6 days	
	Dakar 41667					6 days	
	Dakar 41519					6 days	
ZIKV	FSS13025	3, C57BL/6	1×10^5^ pfu	SC	100%	6–7 days	Rossi et al., 2016
		5, C57BL/6			50%	8–9 days	
		11, C57BL/6			0%	N/A	
EBOV	Mayinga	8–16, 129	10^3^ pfu	SC	100%	7.3 days	Bray, 2001
				IP		5.4 days	
	Kikwit				0%	N/A	
SUDV	Boneface				100%	6.3 days	
RESTV					0%	N/A	
TAFV					0%	N/A	
MARV	Musoke				67%	8.5 days	
RAVV					100%	6.0 days	
MARV	Popp	6–9, 129	10^2.8^ TCID_50_	Aerosol	100%	13.0 days	Lever et al., 2012
			10^3.8^ TCID_50_			12.0 days	
			10^4.8^ TCID_50_			10.2 days	
			10^5.8^ TCID_50_			11.0 days	
EBOV	E719		10^0^ TCID_50_			8.0 days	
			10^1^ TCID_50_			8.0 days	
			10^2^ TCID_50_			8.0 days	
SUDV	Boneface		10^4.8^ TCID_50_		0%	N/A	
LASV	Josiah	8–12, 129/Sv	10^3^ ffu	IV	0%	N/A	Rieger et al., 2013
	AV						
	BA366						
	Nig04-10						
CCHFV	IbAr2000	7–10, 129 Sv/Ev	10^1^ ffu	IP	100%	4 days	Bereczky et al., 2010
			10^3^ ffu			3 days	
			10^5^ ffu			2 days	
			10^6^ ffu			2 days	
		6–12, C57BL/6	10^4^ TCID_50_	IP	100%	4 ± 0 days	Zivcec et al., 2013
CCHFV	IbAr2000	6–12, C57BL/6	10^4^ TCID_50_	IM	100%	5.2 ± 0.6 days	Zivcec et al., 2013
				IN		7 ± 0 days	
				SC		4.6 ± 0.2 days	
SFTSV	YL-1	6–10, 129/Sv	10^6^ ffu	SC	100%	3–4 days	Liu et al., 2014
	SD4	6–12, C57BL/6	10^2^ TCID_50_	IP	100%	5 days	Matsuno et al., 2017
				IM		5–6 days	
				SC		5 days	
				ID		6 days	
			10^5^ TCID_50_	IP		3–4 days	
				IM		4 days	
				SC		4 days	
				ID		6 days	
HeV	SD4	3, C57BL/6	10^6^ pfu	IP	100%	11 days	Dhondt et al., 2013
		6, C57BL/6			83%	11–13 days	
		18, C57BL/6			50%	7 days	
NiV	UMMC1	4–12, C57BL/6	10^2^ pfu		0%	N/A	
			10^3^ pfu		17%	10 days	
			10^4^ pfu		67%	10 days	
			10^5^ pfu		83%	8–10 days	
			10^6^ pfu		100%	6–9 days	
VEEV	V3000	8–12, 129Sv/Ev	1×10^3^ pfu	SC	100%	1 day	Schoneboom et al., 2000
	V3032					1 day	
CHIKV	21	Adult (age not given), 129s/v	20 pfu	ID	100%	3±0.2 days	Couderc et al., 2008
	LR	6–8, C57BL/6	20 ffu	SC	100%	3–4 days	Pal et al., 2013

IM: Intramuscular; IN: Intranasal; SC: Subcutaneous; IP: Intraperitoneal; ID: Intradermal; IV: Intravenous.

**Table 2:** Advantages and disadvantages of using *Ifnar^–/–^* mice for studying human infectious diseases, compared to other strategies

Strategy in small animal models	Advantages	Disadvantages
Knockout mice	Susceptible to a wide range of clinical isolates of viruses	Cannot study immune responses properly due to abnormal innate immunity
	Can study pathogenesis of a new pathogen rapidly	Some viruses may not cause disease in knockout mice
		Cannot test drugs and vaccines effectively
		Can be age-sensitive: older mice may lose their susceptibility to the pathogen
Virus adaptation to host via sequential passaging	Can cause uniform lethality	Not always successful in creating a lethal variant
	Good for screening drugs and vaccines	Can be time consuming to create a lethal variant
	Wild-type mice are widely available	Not clinical isolate of virus and thus may harbour important differences in pathogenesis
Transduction with adenoviral vectors encoding the entry receptor to confer sensitivity	Useful when no other known small animal models exist (i.e., MERS)	Need to know the identity of the receptor
	Can test with clinical isolate of virus	Time consuming to create the recombinant adenovirus

MERS: Middle East respiratory syndrome.

However, *Ifnar^–/–^* mice as a model do have some weaknesses. Since these mice have defective innate immune responses which lead to impaired adaptive immunity, they are not good animal models for studying antiviral compounds, particularly vaccines (Züst et al., 2014). Additionally, it appears that the age of the *Ifnar^–/–^* mice plays a role in host susceptibility to some viruses, as ZIKV loses the ability to cause disease in mice older than six weeks, whereas only partial lethality could be achieved with HeV infection in mice older than six weeks (Table 2). With respect to viruses from the *Coronaviridae* family, the *Ifnar^–/–^* mutation was found instead to prevent the lethal pneumonia observed in SARS-CoV mice (Channappanavar et al., 2016), whereas the absence of the human CD26 (a.k.a. DPP4) receptor for Middle East respiratory syndrome coronavirus (MERS-CoV) means that *Ifnar^–/–^* mice must be first transduced with a human adenovirus serotype 5-vector expressing human CD26 in order to become transiently susceptible to MERS-CoV infection via the IN route (Zhao et al., 2014). The transduced *Ifnar^–/–^* mice were shown to experience ~20% body weight loss and delayed virus clearance by approximately 3 days compared to transduced wild-type mice, but do not die from the infection (Zhao et al., 2014). 

Aside from the transduction strategy, a popular method is to generate host-adapted viruses by sequential passaging in the livers and spleens of rodents (i.e., mice and guinea pigs) *in vivo*, in order to generate increasingly pathogenic virus variants that cause lethal disease to the immunocompetent host. This method has been widely used in the *Filoviridae* field to generate adapted viruses for EBOV (Bray et al., 1998; Volchkov et al., 2000), MARV (Qiu et al., 2014), RAVV (Warfield et al., 2009) and SUDV (Wong et al., 2016) in wild-type mice or guinea pigs. In many cases, these viruses harbour very few mutations compared with the original clinical isolates, and the ability of the adapted virus to evade the host Type I IFN response (via mutations in the viral antigen responsible for this function) is positively correlated with its virulence in the host (Ebihara et al., 2006).

While animal models for studying virus pathogenesis leading to severe disease or lethality should always be the primary priority, an important future aim would be to also establish small animal models for studying pathogen transmission (initial work includes a guinea pig-based model of EBOV transmission (Wong et al., 2015), as well as developing small animal models to study various important phenomena of disease, such as the persistence of ZIKV in the testes of immunocompromised and immunocompetent mice (Govero et al., 2016; Ma et al., 2016), ZIKV infections leading to birth defects in wild-type mice (Cugola et al., 2016), ZIKV infections leading to microcephaly in neonatal mice (Li et al., 2016), or the adulthood sequelae of mice who survived congenital ZIKV infections (Cui et al., 2017). These studies in small animals will set the stage and provide important directives in subsequent investigations of similar disease phenomenon/sequelae in larger animal models, and ultimately, humans.
